# A protocol for high-quality single-cell RNA sequencing with cell surface protein quantification

**DOI:** 10.1097/BS9.0000000000000254

**Published:** 2025-12-31

**Authors:** Sichong Han, Siqi Liu, Changya Chen

**Affiliations:** a State Key Laboratory of Experimental Hematology, National Clinical Research Center for Blood Diseases, Haihe Laboratory of Cell Ecosystem, Institute of Hematology & Blood Diseases Hospital, Chinese Academy of Medical Sciences & Peking Union Medical College, Tianjin 300020, China; b Tianjin Institutes of Health Science, Tianjin 301600, China

**Keywords:** CITE-Seq, Single-cell RNA sequencing

## Abstract

Cellular indexing of transcriptomes and epitopes by sequencing (CITE-seq) enables the simultaneous analysis of transcriptomic and proteomic data at the single-cell level, providing a comprehensive view of cellular heterogeneity and function. In this study, we present a standardized approach for high-quality single-cell RNA sequencing coupled with cell surface protein quantification. Key advantages of CITE-seq include its compatibility with existing scRNA-seq workflows, cost-efficient high-throughput protein detection, and enhanced resolution in cell type classification. Detailed steps for sample preparation, antibody-oligo conjugation, gel bead-in-emulsion (GEM) generation, complementary deoxyribonucleic acid (cDNA) amplification, and library construction are provided, ensuring reproducibility and robust data quality. This protocol facilitates the integration of multimodal single-cell data, enabling precise characterization of rare cell subsets and advancing insights in immunology, oncology, and developmental biology. The workflow is optimized for flexibility across platforms and scalable for diverse research applications.

## 1. INTRODUCTION

Traditional bulk sequencing methods yield average molecular signals derived from heterogeneous cell populations, which inevitably results in the obscuring of rare cell subsets, neglect of cellular heterogeneity, and inability to capture single-cell-specific molecular features. In recent years, the development of single-cell sequencing technologies has enabled the application of genomic, transcriptomic, epigenomic, and multiomic analyses at the single-cell level. These technologies address the limitations of traditional high-throughput sequencing, effectively revealing the gene structure and expression status of individual cells, and hold broad application prospects.^1–3^

Single-cell RNA sequencing (scRNA-seq)^4,5^ involves isolating individual cells, reverse transcribing their RNA into complementary deoxyribonucleic acid (cDNA), amplifying the transcripts, and generating sequencing libraries. The advantages lie in its ability to effectively address cellular heterogeneity,^6–8^ which facilitates the identification of rare cell populations and the delineation of distinct cell subtypes by characterizing gene expression profiles. Additionally, it can be used for the reconstruction of cellular developmental trajectories,^9,10^ and be applied in conjunction with spatial transcriptomics to reveal spatial gene expression characteristics within tissue microenvironments.^11,12^ As the application of scRNA-seq has advanced, its limitations have become increasingly evident:

1. The technology remains relatively complex and costly, hindering its broader use in specific basic research and clinical settings.
2. Commercial single-cell platforms have inherent limitations on cell size, making the technique unsuitable for samples containing large cells.
3. The method cannot simultaneously analyze protein expression. Since proteins are the direct executors of cellular functions, relying solely on RNA-level detection fails to provide a comprehensive understanding of cellular functions and regulatory mechanisms.

Antibody-based single-cell sequencing (Abseq)^13^ is a cutting-edge technology that combines scRNA-seq with high-throughput protein detection, enabling simultaneous analysis of transcriptomes and proteomes at the single-cell level. The core workflow of this technology involves labeling cells with antibodies conjugated to unique DNA oligonucleotides. These antibody-oligos carry barcodes that identify specific proteins, while poly(T)-tailed beads capture both mRNA and antibody-oligos during library preparation. Following reverse transcription and amplification, the combined data are sequenced to enable parallel analysis of RNA and protein expression in thousands of individual cells.^14–16^ Its advantages include:

1. Multimodal data integration: By linking RNA expression to protein activity, Abseq provides a holistic view of cellular states.
2. High-throughput protein profiling: Abseq can analyze hundreds of proteins in a single experiment at a reasonable cost, accelerating the discovery of novel cell subtypes and therapeutic targets.
3. Intracellular protein detection: Abseq supports the analysis of intracellular proteins, expanding its utility in studying cellular signaling pathways and disease pathogenesis.

Cellular indexing of transcriptomes and epitopes by sequencing (CITE-seq),^17^ like Abseq, integrates transcriptomic and proteomic analyses to decode cellular heterogeneity with unprecedented resolution. Developed in 2017, this method combines scRNA-seq with antibody-based protein detection, enabling simultaneous measurement of gene expression and cell surface protein levels in thousands of individual cells—an attribute that makes it invaluable for research in immunology, oncology, and developmental biology.^18–21^ Beyond multimodal data integration and high-throughput protein profiling, its key advantages include:

1. Compatibility with existing workflows: CITE-seq seamlessly integrates with established scRNA-seq pipelines, requiring minimal modifications. This reduces technical barriers and accelerates data generation, making it accessible to labs with preexisting single-cell sequencing infrastructure.
2. Enhanced cell annotation: Protein data from CITE-Seq enhances the accuracy of cell type clustering, particularly in ambiguous cases where RNA signatures alone are insufficient for definitive classification.

While Abseq and CITE-seq share fundamental principles in integrating transcriptomic and proteomic profiling at the single-cell level, CITE-seq demonstrates pronounced advantages in workflow adaptability, analytical versatility, and biological interpretability:

1. Platform agnosticism and workflow interoperability: Abseq is inherently coupled with 10× Genomics’ Chromium platform, imposing constraints on experimental design flexibility and reagent compatibility. In contrast, CITE-seq is engineered for seamless integration with established scRNA-seq pipelines and supports multi-platform deployment. This architectural openness allows researchers to utilize existing infrastructure, minimizing technical barriers and operational costs.
2. Cost-efficient high-throughput proteomic profiling: CITE-seq achieves scalable multiplexed protein analysis at a reduced per-sample cost relative to Abseq. Experimental optimizations, such as antibody titer reduction maintain signal integrity while significantly lowering reagent expenditure. Furthermore, compatibility with sample hashing technologies allows parallel processing of multiple biological replicates within a single run, further enhancing cost-efficiency.
3. Robust data quality and analytical precision: CITE-seq exhibits superior signal-to-noise performance across commercial platforms, which is critical for the identification of rare cell subsets, such as therapy-resistant cancer stem cells or dysfunctional exhausted T cells defined by low-abundance surface markers.

Despite inherent technical limitations-including restriction to cell surface protein detection, signal-to-noise ratios contingent on antibody titration efficiency, and more stringent constraints on cell size-CITE-seq exhibits broader applicability relative to Abseq. This characteristic positions it as a more valuable tool for elucidating disease mechanisms and identifying potential therapeutic targets in single-cell multiomic analyses. Subsequent sections will provide a detailed description of the specific experimental procedures inherent to CITE-seq.

## 2. MATERIALS

### 2.1. Equipment

**Table:** 

Item	Description	Company	Cat. no.
0.2 mL PCR 8-tube strips	PCR tubes 0.2 mL 8-tube strips	Eppendorf	951010022
	TempAssure PCR 8-tube strip	USA Scientific	1402-4700
	MicroAmp 8-tube strip, 0.2 mL	Thermo Fisher Scientific	N8010580
	MicroAmp 8-Cap Strip, clear		N8010535
1.5 mL tubes	DNA LoBind Tubes 1.5 mL	Eppendorf	22431005
2 mL tubes	DNA LoBind Tubes 2 mL		22431048
15 mL tubes	15 mL centrifuge tubes	Corning	CLS430791
50 mL tubes	50 mL centrifuge tubes		CLS430829
Filters	MACS SmartStrainers, 30 µm	Miltenyi Biotec	130-098-458
	Flowmi Cell Strainer, 40 µm	Bel-Art Flowmi	H13680-0040
	Flowmi Cell Strainer, 70 µm		H13680-0070
Vortex	Vortex Mixer	VWR	10153-838
Centrifuge	Refrigerated Eppendorf Centrifuge or any equivalent centrifuge	Millipore Sigma	5427R or 5424R
Sorter	MA900 Multi-Application Cell Sorter or any equivalent cell sorter	Sony	MA900
-	Inverted tissue culture microscope with 10×/20× magnification and fluorescence imaging capability	-	-
Cell counter and slides-Countess	Countess II FL Automated Cell Counter	Thermo Fisher Scientific	AMQAF1000
	Countess 3 FL Automated Cell Counter		A49866
	Countess Cell Counting Chamber Slides		C10228
Cell counter and slides-Luna	LUNA-FL Automated Fluorescence Cell Counter	Logos Biosystems	L20001
	LUNA Cell Counting Slides, 50 Slides		T13001
Cell counter and slides-Cellometer	Cellometer K2 Bundle w/Matrix Software	Nexcelom Bioscience	CMT-K2-MX-150
	PD100 Counting Chambers 1 case		CHT4-PD100-003
Cell counter and slides-NucleoCounter	NucleoCounter NC-202 Instrument	ChemoMetec	970-2020
	Via2-Cassette		941-0024
Cell counter and slides-ViCELL BLU	Vi-CELL BLU System	Beckman Coulter	C19201
	Sample vials		C24843
Biometra TAdvanced 96 SG/S*	-	Analytik Jena	846-x-070-241/846-x-070-251
Mastercycler X50s/X50a**	-	Eppendorf	6311000010/6313000018
VeritiPro***	-	ThermoFisher	A48141
Veriti 96-Well Thermal Cycler (discontinued)	-		4375786
PTC Tempo Deepwell	-	Bio-Rad	12015392
C1000 Touch Thermal Cycler with 96-Deep Well Reaction Module (discontinued)	-		1851197
Mastercycler Pro (discontinued)	-	Eppendorf	North America 950030010 International 6321 000.019
Vortex Mixer	Vortex Mixer	VWR	10153-838
-	Divided Polystyrene Reservoirs		41428-958
Centrifuge	VWR Mini Centrifuge		76269-066
-	Eppendorf ThermoMixer C	Eppendorf	5382000023
-	Eppendorf SmartBlock 1.5 mL, Thermoblock for 24 reaction vessel		5360000038

### 2.2. Reagents

**Table:** 

Reagent	Company	Cat. no.
Trypan Blue Label (0.4%)	Thermo Fisher Scientific	T10282
Live/Dead Viability/Cytotoxicity Kit for mammalian cells		L3224
Acridine orange/propidium iodide stain	Logos Biosystems	F23001
ViaStain AO/PI (acridine orange/propidium iodide)	Nexcelom Bioscience	CS2-0106-5mL
VS Cellometer AOPI Staining Solution		CS2-0106-25mL
Vi-CELL BLU single reagent kit	Beckman Coulter	C06019
0.5 M single-use concentration control (20 vials of 0.5 × 10^6^ beads/mL)		C09147
2.0 M single-use concentration control (20 vials of 2 × 10^6^ beads/mL)		C09148
4.0 M single-use concentration control (20 vials of 4 × 10^6^ beads/mL)		C09149
10.0 M single-use concentration control (20 vials of 10 × 10^6^ beads/mL)		C09150
50% single-use viability control (20 vials of 50% viability beads)		C09145
Phosphate-buffered Saline without Calcium and Magnesium	Corning	21-040-CM
Molecular Grade Nuclease-free Water	Thermo Fisher Scientific	AM9937
Fetal Bovine Serum, qualified, heat inactivated	Thermo Fisher Scientific	16140071
Avantor Seradigm Premium Grade Fetal Bovine Serum	VWR	97068-085
Invitrogen eBioscience 7-AAD Viability Staining Solution	Invitrogen	00699350
UltraPure Bovine Serum Albumin (BSA, 50 mg/mL)	Thermo Fisher Scientific	AM2616
MACS BSA Stock Solution	Miltenyi Biotec	130-091-376
Albumin, Bovine Serum, 10% Aqueous Solution, Nuclease-Free For isolation of cells from FFPE tissue sections	Millipore Sigma	126615
Bovine serum albumin in DPBS (10%)		A1595
Oligonucleotide Conjugation Kit	Abcam	ab218260
Custom DNA Oligos	IDT	-
100 μg purified azide-free antibody (1 mg/mL)	-	-
Human TruStain FcX (Fc Receptor Blocking Solution)	BioLegend	422301
TotalSeqTM antibody-oligonucleotide conjugates		-
Cell Staining Buffer		420201
Antibodies (Fluorophore) if using FACS for enriching labeled cells		-
UltraPure bovine serum albumin (BSA, 50 mg/mL)	Thermo Fisher ScientificMillipore Sigma	AM2616
Trypan Blue Stain (0.4%)		T10282
Bovine serum albumin in DPBS (10%) alternative to Thermo Fisher product		A1595
MACS BSA Stock Solution alternative to Thermo Fisher product	Miltenyi Biotec	30-091-376
Phosphate-buffered saline, 1× without Calcium and Magnesium	Corning	21-040-CV
Fetal bovine serum alternative to Thermo Fisher product	VWR	97068-085
DNA LoBind Tube 5 mL	EppendorfThermo Fisher Scientific	0030108310
Low TE Buffer (10 mM Tris-HCl pH 8.0, 0.1 mM EDTA)	Millipore Sigma	12090-015
Ethanol, Pure (200 Proof, anhydrous)		E7023-500ML
SPRIselect Reagent Kit	Beckman Coulter	B23318
10% Tween 20	Bio-Rad	1662404
Glycerin (glycerol), 50% (v/v) Aqueous Solution	Ricca Chemical Company	3290-32
Qiagen Buffer EB	Qiagen	19086
2100 Bioanalyzer Instrument & Laptop Bundle	Agilent	G2939BA & G2953CA
High-sensitivity DNA Kit		5067-4626
4200 TapeStation		G2991AA
High Sensitivity D1000 ScreenTape/Reagents		5067-5584/5067-5585
High Sensitivity D5000 ScreenTape/Reagents		5067-5592/5067-5593
Fragment Analyzer Automated CE System - 12 cap	Advanced Analytical	FSv2-CE2F
Fragment Analyzer Automated CE System - 48/96 cap		FSv2-CE10F
High Sensitivity NGS Fragment Analysis Kit		DNF-474
LabChip GX Touch HT Nucleic Acid Analyzer	PerkinElmer	CLS137031
DNA High Sensitivity Reagent Kit		CLS760672
KAPA Library Quantification Kit for IlluminaPlatforms	KAPA Biosystems	KK4824

**Caution:** This list may not include some standard laboratory equipment.

For some items, a number of vendor options are listed. Choose item based on availability and preference.

For select instruments, ramp rates should be adjusted for all steps as described: *Analytik Jena Biometra TAdvanced 96 SG/S: 2°C/s for both heating and cooling.

**Eppendorf Mastercycler X50s/X50a: 3°C/s heating and 2°C/s cooling.

***ThermoFisher VeritiPro requires FW 1.2.0, 96-well tray/retainer (PN 4381850), and “Cover Ramping” enabled.

### 3. PROCEDURE

**Figure 1.**
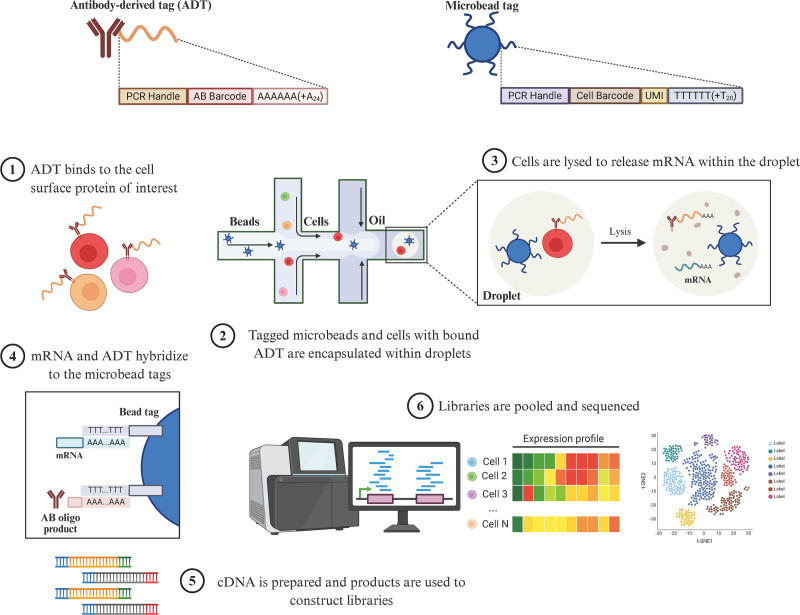
The workflow of the entire experimental process.

The workflow chart illustrating the entire experimental process is shown in Figure 1.

**1. Sample preparation**

Overview of the recommended wash and resuspension buffers for fresh cells

**Table:** 

Fresh cells	General sample preparation	Feature-specific sample preparation protocols	
		Cell surface protein	Barcode enabled antigen mapping labeling
Human and mouse cell lines (cryopreserved or freshly cultured)	Phosphate-buffered saline (PBS) + 0.04% BSA	PBS + 1% BSA/PBS + 10% FBS/cell culture media + 1% BSA	-
Human blood cells (PBMCs, BMMCs)	PBS + 0.04% BSA	PBS + 1% BSA/PBS + 10% FBS	PBS + 2% FBS
Mouse blood cells (PBMCs, BMMCs, splenocytes)	PBS + 1% BSA	PBS + 1% BSA/PBS + 10% FBS	PBS + 2% FBS
Dissociated tumor cells (DTCs)	Cell Culture Media + 10% FBS	PBS + 1% BSA/PBS + 10% FBS	-

Overview of the recommended cell strainer

**Table:** 

Strainer	Size and bore	Average volume loss	Average change in concentration
MACS SmartStrainer	30 µm, wide	130.0 µL ± 36.0 µL	11% ± 3%
Flowmi Cell Strainer	40 µm, regular	20.0 µL ± 9.4 µL	21% ± 6%

**Caution:** For general use, the MACS SmartStrainer is recommended. It generally causes minimal changes to cell concentration. A minimum sample volume of >600 µL is required due to volume loss that can occur. For low cell suspension volumes, the Flowmi Cell Strainer is recommended.

Due to some volume loss during filtering, always recount the cell suspension after straining.

**1.1 Sample preparation (for abundant cells) timing: ~30 minutes**

(1) Obtain the cells of interest in suspension from cell culture, dissociated tissues, cell thawing, or other cell isolation methods.
(2) Using a wide-bore pipette tip, gently and thoroughly mix the cells, then determine the cell concentration using an automated cell counter or a hemocytometer.
(3) Calculate the cell resuspension volume required for the desired target concentration (assume about 50% cell loss). If significant amounts of cell clumps or debris are observed, gently mix cells by pipetting up and down 10 to 15 times and filter cells using an appropriate cell strainer.
(4) Centrifuge cells at 300× *g* for 5 minutes at room temperature and remove the supernatant without disrupting the cell pellet.

**Caution:** Smaller or larger cells may require optimization of centrifugation conditions. Speed and time may be adjusted to minimize cell loss and preserve cell integrity. Know the expected position of the pellet, especially when working with small or limited cells, as the pellet can be difficult to see.

(5) Add 1 mL phosphate-buffered saline (PBS) with 0.04% to 1% BSA or 10% FBS and repeat step 4.
(6) Add the appropriate volume (as determined at step 3) of 1× PBS with 0.04% to 1% BSA or 10% FBS to the tube. Gently pipette 5 to 10 times or until the cells are completely suspended.
(7) Determine the cell concentration using a manual/automated cell counter. If cell aggregates/clumps are observed, filter the sample using a cell strainer and recount.

**Caution:** If cell concentration is significantly lower than expected, the sample may not have formed efficient cell pellets during centrifugation. Check the concentration of the saved supernatants and re-pellet at a higher centrifugation speed.

Cell loss may be the result of non-optimized cell buffer resulting in lysis or death, or occur during filtering steps.

(8) Adjust the volume to obtain the target cell concentration, if necessary and recount. Once the target cell concentration is obtained, place the cells on ice and proceed to the next step.

**1.2 Sample preparation (for limited cells) timing: ~30 minutes**

**Caution:** The protocol described here is best suited if the initial cell suspension has <200,000 cells in total (<200,000 suspension or <100,000 adherent cells).

(1) Obtain the cells of interest in suspension from cell culture, dissociated tissues, cell thawing, or other cell isolation methods.
(2) Using a wide-bore pipette tip, gently and thoroughly mix the single-cell suspension. If feasible, determine the cell concentration using an automated cell counter or a hemocytometer.
(3) If cell clumps or cell debris are observed, gently mix cells by pipetting up and down 10 to 15 times and filter cells using an appropriate cell strainer.
(4) Centrifuge cells at 300× *g* for 5 minutes.

**Caution:** Some cells need to be centrifuged at higher speed (eg, up to 400× *g*) or for longer time (eg, up to 10 minutes) to minimize cell loss due to inefficient pelleting.

Know the expected position of the pellet, especially when working with small or limited cells, as the pellet can be difficult to see.

(5) Remove the supernatant without disrupting the cell pellet, leaving an appropriate volume to achieve the target cell concentration. Save the removed supernatant in another tube until the protocol is complete.
(6) If performing a wash step, add 1 mL PBS with 0.04% to 1% BSA or 10% FBS, and repeat steps 3 to 4. One cell wash is recommended to remove any ambient RNA and contaminants.
(7) Using a regular-bore pipette tip, resuspend the cell pellet in the leftover supernatant by gently pipetting up and down 10 to 15 times.
(8) Determine the cell concentration using an automated cell counter or a hemocytometer, and if necessary, adjust the volume to obtain the target cell concentration.
(9) Once the target cell concentration is obtained, place the cells on ice and proceed to the next step.

**2. Cell surface protein labeling**

**2.1 Antibody-oligonucleotide conjugation timing: ~2 hours**

**Caution:** Both single- and double-stranded oligos can be conjugated. A single-stranded oligo must be 10 to 120 nt long and contain a terminal amine group, which must be added during synthesis. (All commercial oligo suppliers offer this modification.) The efficiency of conjugation is slightly higher with 5’ aminated oligos. Double-stranded oligos can be up to 80 bp in length but only one end should be aminated.

The oligo must be purified by HPLC, at a concentration of 60 to 100 μM in 100 μL of a suitable buffer (Table 2.1.1). If the oligo concentration is greater than 100 μM, dilute to 100 μM in wash buffer.

For optimal results, the antibody must be purified and at a concentration of 1 mg/mL in 100 μL of a suitable buffer (Table 2.1.1). Higher antibody concentrations should be diluted to 1 mg/mL with wash buffer.

**Table 2.1.1.** Buffer considerations

Buffer component	Oligo buffer	Antibody buffer
pH	6-8	7-9
Amine-free buffer (ideally phosphate buffer)	√	√
Non-buffering salts (eg, sodium chloride)	√	√
Chelating agents (eg, EDTA)	√	√
Sugars	√	√
Glycerol	<50%	<50%
Thiomersal (Thimerosal, Methiolate)	×	×
Sodium azide*	<0.1%	<0.1%
BSA*	<0.1%	<0.1%
Gelatin*	<0.1%	<0.1%
Tris	×	<20 mM
Glycine	×	×
Primary amines	×	×
Thiols (eg, mercaptoethanol or DTT)	×	×

* Individually, the concentrations shown for these components should not affect the reaction. However, in combination with other compounds that are not recommended above a certain concentration, the reaction may be affected.

2.1.1 Oligo activation: Add 100 μL of the oligo to the Oligo Activation Reagent vial. Mix gently and incubate for 30 minutes at room temperature. Proceed to step 2 during the incubation.
2.1.2 Antibody activation: Add 100 μL of the antibody (at 1 mg/mL concentration) to the Antibody Activation Reagent vial. Mix gently and incubate for 30 minutes at room temperature. Proceed to step 3 during the incubation.
2.1.3 Desalting of activated material:
  (1) Secure each separating column in a vertical position. Remove the upper cap first. Then remove the lower cap and allow the storage liquid to flow through the column. Discard the column flow-through.
  (2) Equilibrate each column by adding 3 mL of wash buffer to the top of the column and allowing the liquid to flow through under gravity. Discard the flow-through. Repeat a further 4 times.
  (3) After the 30-minute incubation for activation in steps 1 and 2, add 100 μL of activated oligo or antibody to the top of the column and allow the liquid to completely absorb into the column. Collect the flow through and keep until confirmed successful conjugation.
  (4) Add 550 μL of wash buffer to the top of the column. This will push the activated material to the bottom of the column. Allow this liquid to completely absorb before proceeding to the next step. Collect the flow through and keep until confirmed successful conjugation.
  (5) Place a clean microcentrifuge tube under the column. Add 300 µL of wash buffer to the top of the column.
  (6) Collect the eluate from the column. This column eluate (300 µL) contains activated oligo or antibody that is ready for use in conjugation.
2.1.4 Storage of activated material:
  (1) Oligo and control oligo: The activated oligo can be stored at room temperature for up to 8 hours. For long-term storage of up to 12 months, storage at −20°C is recommended.
  (2) Antibody and control antibody: The activated antibody should be stored on ice and used within 2 hours. The activated antibody is not stable enough for long-term storage.
2.1.5 Generation of purified antibody-oligo conjugate:
  (1) Add 300 μL of activated antibody to the appropriate volume of activated oligo and wash buffer as shown in Table 2.1.2.

**Table 2.1.2.** Antibody:oligo ratios

Volume of activated antibody (μL)	Volume of activated oligo (μL)	Volume of wash buffer (μL)	Antibody:oligo starting molar ratio
300	300	0	1:15
300	200	100	1:10
300	100	200	1:5
300	60	240	1:3

**Caution:** The preferred ratio will depend upon the experiment that the conjugate will be used in and may need to be determined experimentally.

(2) Mix and incubate at room temperature for 1 hour. Conjugations can also be incubated overnight at room temperature with no adverse effect.
(3) The conjugate is now ready for use, and may be purified to remove any unbound oligos if this is required for application (see step 6).
(4) Any unused activated oligo may be stored at −20°C.

2.1.6 Conjugate purification:
**Caution:** 50 µg of antibody is the lower limit for seeing a clearly visible pellet.
  (1) Warm the Conjugate Clean Up Reagent by placing the tube in warm water (not warmer than 40°C) for 10 minutes and mixing regularly. If the sample does not dissolve completely, spin the sample in a bench-top microcentrifuge at a recommended maximum speed of 13,000× *g* for 1 minute, and use the supernatant.
  (2) Add an equal volume of Conjugate Clean Up Reagent to the volume of antibody/oligo mixture, mix, and incubate at room temperature or on ice for 20 minutes. For example, add 600 µL of conjugate clean-up reagent to 600 µL of antibody/oligo mixture.
  (3) Centrifuge in a bench-top microcentrifuge for 5 minutes at 15,000× *g*. The required spin time will vary depending on buffer composition and speed. The speed should not exceed 15,000× *g*. Position the Eppendorf tube in the centrifuge in such a manner that you know where your pellet will be located.
  (4) Remove the sample from the centrifuge, taking care not to dislodge the small pellet at the bottom of the tube. If no pellet is seen, add more Conjugate Clean Up Reagent (another 1/10 volume), mix well, and incubate on ice for a further 10 minutes, and centrifuge. If no pellet is seen when using a protein other than an antibody, add half of the volume of Conjugate Clean Up Reagent added at step (2), mix well, and incubate on ice for a further 10 minutes and centrifuge. For example, if 600 µL of Conjugate Clean Up Reagent were added at step (2), top up with another 300 µL.
  (5) Carefully remove the supernatant and store until efficient precipitation has been confirmed.
  (6) Add 100 µL of the Antibody Suspension Buffer to the pellet and mix gently.
  (7) To remove as much free oligo as possible, a second cleanup should be performed on the same conjugate.
  (8) The antibody/oligo conjugate is now ready to use.
2.1.7 Use of control oligo and antibody:
**Caution:** A control oligo (a 30 bp oligo with a 5’ terminal amine) and a control antibody (rabbit IgG) are used as positive controls to give the option of confirming the conjugation chemistry is working optimally.
  (1) Add 100 µL of wash buffer to both the lyophilized vials of control oligo and control antibody.
  (2) Add the 100 µL of control oligo to a vial of Oligo Activation Reagent. Mix and incubate at room temperature for 30 minutes.
  (3) Add the 100 µL of control antibody to a vial of Antibody Activation Reagent. Mix and incubate at room temperature for 30 minutes.
  (4) Meanwhile, proceed to the desalting procedure (step 3).
2.1.8 Conjugate storage: To maximize stability, the conjugate is recommend storing in a form that is as concentrated as possible and at a low temperature. Checking with the antibody and oligo manufacturers if their products can be stored in 50% glycerol at −20°C, most conjugates can be stored in these conditions, as long as they are compatible with the unconjugated antibody and oligo. If it is appropriate for your reagents and subsequent experiments, the addition of preservatives may also be helpful.
2.1.9 Analysis of the antibody-oligo conjugate:
  (1) A small amount (5–10 μg) of the conjugate can be run on a reducing SDS-PAGE gel. Mix the conjugate sample with 2× gel loading buffer and heat at 100°C for 5 minutes.
  (2) Cool the sample, then load onto a reducing SDS-PAGE gel. A 4% to 12% gradient gel is recommended for best results. Run the gel under reducing conditions and stain for protein using Coomassie Blue stain or a suitable equivalent. After destaining, the gel can be analyzed for the presence of antibody-oligo conjugates.

**Caution:** Apart from custom conjugated antibodies introduced above, preconjugated antibodies BioLegend TotalSeq™-B or BioLegend TotalSeq™-C is another option.

**2.2 Cell surface protein labeling timing: ~1 hour**

**Caution:** Use distinct antibody clones for fluorescence-activated cell sorting (FACS) and cell surface protein labeling. Optimize working concentration of each of the antibodies used.

This protocol was demonstrated using 0.2 to 2 × 10^6^ cells. All steps can be performed in 5 mL centrifuge tubes, 15 mL centrifuge tubes, or round-bottom FACS tubes.

**Figure 2.**
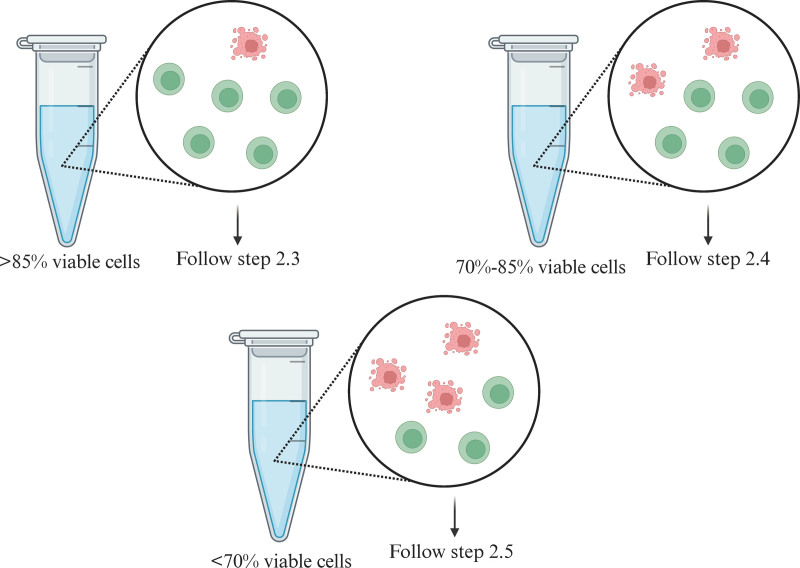
Sample type-specific washing protocols.

1 Determine cell viability of the sample as cell washing instructions are different for samples with different cell viability (**Fig. 2**).

2.2.2 Prepare buffers:
(1) For samples containing >70% viable cells: chilled (4°C) PBS + 1% BSA, PBS + 0.04% BSA.
(2) For samples containing <70% viable cells: chilled (4°C): PBS + 10% FBS.
2.2.3 Prepare antibody mix supernatant:
  (1) Add appropriate/manufacturer’s recommended amount of antibody-oligonucleotide conjugates to a 1.5 mL microcentrifuge tube. If using a custom lyophilized antibody, resuspend the antibody-oligonucleotide conjugates in an appropriate volume of labeling buffer.
  (2) Centrifuge the mix at 14,000× *g* for 10 minutes at 4°C.
  (3) Transfer the supernatant (containing antibody mix) to a new tube and maintain at 4°C.
  2.2.4 Prepare FACS antibody pool:
    (1) Add appropriate/manufacturer’s recommended amount of fluorophore antibodies to a 1.5 mL microcentrifuge tube on ice.
    (2) Gently pipette mix and maintain at 4°C. Avoid light exposure.
  2.2.5 Transfer cells to a new 5 mL tube and add chilled PBS + 0.04% BSA for a total 1 mL volume. For samples containing <70% viable cells, use chilled PBS + 10% FBS.
  2.2.6 Centrifuge cells at 4°C. Use of swinging-bucket rotor is recommended for higher cell recovery. Centrifugation speed and time depend upon the sample type (Table 2.2.1). Larger or fragile cell types may require slower centrifugation speeds.
  2.2.7 Remove the supernatant without disturbing the pellet and resuspend cell pellet in 50 μL chilled PBS + 1% BSA. For samples containing <70% viable cells, resuspend in chilled PBS + 10% FBS.
  2.2.8 Add 5 μL Human TruStain FcX. Gently pipette mix and incubate for 10 minutes at 4°C.
  2.2.9 Add the prepared antibody mix supernatant. If also performing FACS enrichment, add FACS antibody pool.
  2.2.10 Add chilled PBS + 1% BSA to the cells to bring the total volume to 100 μL. Pipette set to 90 μL to gently mix 10 times. For samples containing <70% viable cells, add chilled PBS + 10% FBS.
  2.2.11 Incubate for 30 minutes at 4°C. If using FACS antibodies, incubate without light exposure. Proceed to cell washing.

**Caution:** Cell washing protocol and wash buffers depend upon the sample. Choose an appropriate protocol based on the starting sample viability.

**Table 2.2.1:** Sample type specific centrifugation conditions

Sample type	Centrifugation conditions
Samples containing >85% viable cells, eg, PBMCs	300–400× *g* for 5 min
Samples containing <85% viable cells, eg, tumor cells	150–300 × *g* for 5–10 min

**2.3 Cell washing (for samples with >85% viable cells) timing: ~1–2 hours**

2.3.1 Wash by adding 3.5 mL chilled PBS + 0.04% BSA to the labeled cells. Gently pipette mix.
2.3.2 Centrifuge at 4°C. Centrifugation speed and time depend upon the sample type (Table 2.2.1).
2.3.3 Remove the supernatant without touching the bottom of the tube to avoid dislodging the pellet, leaving behind excess supernatant may cause non-specific binding, which may result in increased background reads during sequencing.
2.3.4 Resuspend the pellet in 100 μL room temperature PBS and transfer to a new 5 mL tube. Incubate for 5 minutes at room temperature.
2.3.5 Using a pipette tip, resuspend the pellet or cells in 3.5 mL chilled PBS + 1% BSA.
2.3.6 Centrifuge at 4°C. Centrifugation speed and time depend upon the sample type (Table 2.2.1) and remove the supernatant without touching the bottom of the tube to avoid dislodging the pellet.
2.3.7 Repeat steps 5 to 6 two times for a total of 4 washes.
2.3.8 For enrichment of labeled and viable cells by FACS:
  (1) Based on starting concentration and assuming about 50% cell loss, add an appropriate volume chilled PBS + 2% FBS (including a dead cell marker) to obtain a final cell concentration of 5–10 × 10^6^ cells/mL.
  (2) Proceed to FACS, and determine cell concentration and viability using a Countess II Automated Cell Counter or a hemocytometer.
  (3) Proceed immediately to step 3.

If not performing FACS:

(1) Based on starting concentration and assuming about 50% cell loss, add an appropriate volume of chilled PBS + 1% BSA to obtain a concentration of 700 to 1200 cells/μL.
(2) Determine cell concentration and viability using a Countess II Automated Cell Counter or a hemocytometer.
(3) Proceed immediately to step 3.

**2.4 Cell washing (for samples with 70% to 85% viable cells) timing: ~1–2 hours**

2.4.1 Wash by adding 3.5 mL chilled PBS + 1% BSA to the labeled cells. Gently pipette mix.
2.4.2 Centrifuge at 4°C. Centrifugation speed and time depend upon the sample type (Table 2.2.1).
2.4.3 Remove the supernatant without touching the bottom of the tube to avoid dislodging the pellet.
2.4.4 Using a pipette tip, resuspend the pellet or cells in 3.5 mL chilled PBS + 1% BSA and transfer to a new 5 mL tube.
2.4.5 Centrifuge at 4°C. Centrifugation speed and time depend upon the sample type (Table 2.2.1).
2.4.6 Remove the supernatant without touching the bottom of the tube to avoid dislodging the pellet.
2.4.7 Using a pipette tip, resuspend the pellet or cells in 3.5 mL chilled PBS + 1% BSA.
2.4.8 Centrifuge at 4°C. Centrifugation speed and time depend upon the sample type (Table 2.2.1).
2.4.9 Remove the supernatant without touching the bottom of the tube to avoid dislodging the pellet.
2.4.10 For enrichment of labeled and viable cells by FACS:
  (1) Based on starting concentration and assuming about 50% cell loss, add an appropriate volume chilled PBS + 2% FBS (including a dead cell marker) to obtain a final cell concentration of 5–10 × 10^6^ cells/mL and proceed to FACS.
  (2) Determine cell concentration and viability using a Countess II Automated Cell Counter or a hemocytometer.
  (3) Proceed immediately to step 3.
If not performing FACS:
  (1) Based on starting concentration and assuming about 50% cell loss, add an appropriate volume of chilled PBS + 1% BSA to obtain a concentration of 700–1200 cells/μL.
  (2) Determine cell concentration and viability using a Countess II Automated Cell Counter or a hemocytometer.
  (3) Proceed immediately to step 3.

**2.5 Cell washing (for samples with <70% viable cells) timing: ~1–2 hours**

2.5.1 Wash by adding 3.5 mL chilled PBS + 10% FBS to the labeled cells. Gently pipette mix.
2.5.2 Centrifuge at 4°C. Centrifugation speed and time depend upon the sample type (Table 2.2.1).
2.5.3 Remove the supernatant without touching the bottom of the tube to avoid dislodging the pellet.
2.5.4 Using a pipette tip, resuspend the pellet or cells in 3.5 mL chilled PBS + 10% FBS and transfer to a new 5 mL tube.
2.5.5 Centrifuge at 150 to 300× *g* for 5 to 10 minutes at 4°C. Centrifugation speed and time depend upon the sample type (Table 2.2.1), larger or fragile cell types may require slower centrifugation speeds.
2.5.6 Remove the supernatant without touching the bottom of the tube to avoid dislodging the pellet.
2.5.7 Using a pipette tip, resuspend the pellet or cells in 3.5 mL chilled PBS + 10% FBS.
2.5.8 Centrifuge at 150 to 300× *g* for 5 to 10 minutes at 4°C. Centrifugation speed and time depend upon the sample type (Table 2.2.1).
2.5.9 Remove the supernatant without touching the bottom of the tube to avoid dislodging the pellet.
2.5.10 For enrichment of labeled and viable cells by FACS:
  (1) Based on starting concentration and assuming about 50% cell loss, add an appropriate volume chilled PBS + 10% FBS (including a dead cell marker) to obtain a final cell concentration of 5 to 10 × 10^6^ cells/mL and proceed to FACS.
  (2) Determine cell concentration and viability using a Countess II Automated Cell Counter or a hemocytometer.
  (3) Proceed immediately to step 3.
If not performing FACS:
  (1) Based on starting concentration and assuming about 50% cell loss, add an appropriate volume of chilled PBS + 10% FBS to obtain a concentration of 700 to 1200 cells/μL.
  (2) Determine cell concentration and viability using a Countess II Automated Cell Counter or a hemocytometer.
  (3) Proceed immediately to step 3.

**Figure 3.**
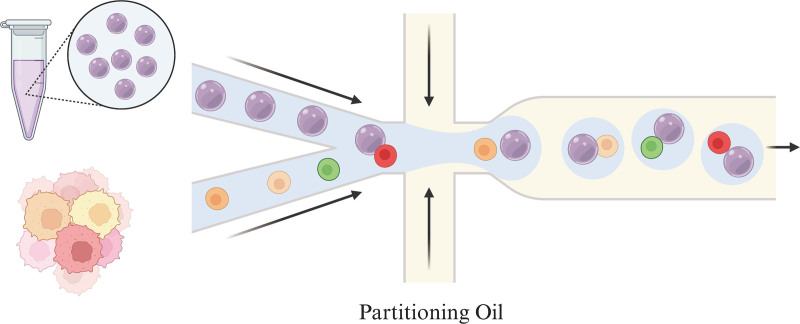
GEM generation and barcoding overview. GEM = gel bead-in-emulsion.

**3. Gel bead-in-emulsion (GEM) generation and barcoding (Fig. 3**)

**3.1 Prepare mix and run the machine timing: 48 minutes**

(1) Prepare reaction mix on ice. Pipette mix 15 times and centrifuge briefly.
(2) Add a proper volume of mix into each tube of a PCR 8-tube strip on ice.
(3) Assemble the Chip and add 50% glycerol solution to each unused well if processing <8 samples per Chip.
(4) Add the appropriate volume of nuclease-free water to reaction mix according to the cell stock concentration and pipette 5 times. Add the corresponding volume of single-cell suspension to mix and dispense reaction mix plus cell suspension into the bottom center of each well in row 1 without introducing bubbles.
(5) Vortex gel beads for 30 seconds and centrifuge for about 5 seconds, then slowly aspirate an appropriate amount of gel beads into the wells in row 2 without introducing bubbles, wait 30 seconds.
(6) Dispense partitioning oil into the wells in row 3 without dripping into other wells.
(7) Run the Chip in the Machine after ensuring that the Chip stays horizontal.

**3.2 GEM-RT incubation timing: 58 minute-STOP POINT**

(1) Pre-cool by placing a tube strip on ice in advance.
(2) Slowly aspirate GEMs from the lowest points of the recovery wells in row 3 without creating a seal between the tips and the bottom of the wells.
(3) Dispense GEMs into the tube strip on ice within 20 seconds.
(4) Incubate in the thermal cycler which can accommodate at least 100 µL volume with the following protocol:

**Table:** 

Lid temperature	Reaction volume	Run time
53°C	125 µL	~55 min
Step	Temperature	Time
1	53°C	00:45:00
2	85°C	00:05:00
3	4°C	Hold

Then the product can be stored at 4°C for up to 72 hours or at −20°C for up to a week, or proceed to the next step.

**4. Post-GEM-RT cleanup and cDNA amplification**

**4.1 Post-GEM-RT cleanup-dynabeads timing: 45 minutes**

(1) Add a proper volume of Recovery Agent to each sample at room temperature. Do not pipette mix or vortex the biphasic mixture. Wait 2 minutes.

**Caution:** A smaller aqueous phase volume indicates a clog during GEM generation.

(2) Slowly remove and discard recovery agent/partitioning oil from the bottom of the tube. Do not aspirate any aqueous sample.
(3) Prepare dynabeads cleanup mix:

**Table:** 

Dynabeads cleanup mix	Cleanup buffer	Dynabeads MyOne SILANE	Reducing agent B	Nuclease-free water	Total
1× (μL)	182	8	5	5	200

**Caution:** The Dynabeads MyOne SILANE needs to be vortexed thoroughly more than 30 seconds immediately before adding to the mix. Aspirate the full liquid volume with a pipette tip to verify that the beads have not settled in the bottom of the tube. If clumps are present, pipette mix to resuspend completely. Do not centrifuge before use.

(4) Vortex and add 200 µL to each sample. Pipette set to 200 µL and mix 10 times.
(5) Incubate 10 minutes at room temperature (keep caps open). Pipette mix again at about 5 minutes after the start of incubation to resuspend settled beads.
(6) Prepare elution solution I, vortex, and centrifuge briefly:

**Table:** 

Elution solution I	Buffer EB	10% Tween 20	Reducing agent B	Total
1× (μL)	98	1	1	100

(7) At the end of 10-minute incubation, place on a magnetic separator•High position until the solution clears.
(8) Remove the supernatant, and add 300 µL 80% ethanol to the pellet while on the magnet, wait 30 seconds.
(9) Remove the ethanol, and add 200 µL 80% ethanol to pellet, wait 30 seconds.
(10) Remove the ethanol and centrifuge briefly. Then place on the magnet•Low.
(11) Remove remaining ethanol and air dry for 1 minute. Remove from the magnet and immediately add 35.5 µL elution solution I (prepared in step 4.1.6).
(12) Pipette set to 30 µL and mix without introducing bubbles, and incubate 2 minutes at room temperature.
(13) Place on the magnet•Low until the solution clears, and transfer 35 µL sample to a new tube strip.

**4.2 cDNA amplification timing: 40 minute-STOP POINT**

(1) Prepare cDNA amplification mix on ice. Vortex and centrifuge briefly:

**Table:** 

cDNA amplification reaction mix	Amp mix	Feature cDNA primers 2	Total
1× (µL)	50	15	65

(2) Add 65 µL cDNA amplification reaction mix to 35 µL sample, pipette set to 90 µL to mix and centrifuge briefly.
(3) Incubate in a thermal cycler with the following protocol:

**Table:** 

Lid temperature	Reaction volume	Run time
105°C	100 µL	~30–45 min
Step	Temperature	Time
1	98°C	00:03:00
2	98°C	00:00:15
3	63°C	00:00:20
4	72°C	00:01:00
5	Go to step 2, check Table 4.2.1 for the number of cycles	
6	72°C	00:01:00
8	4°C	Hold

**Table 4.2.1:** Recommended starting point for cycle number optimization

Targeted cell recovery	Total cycles
<500	13
500–6000	12
>6000	11

**Caution:** The optimal number of cycles is a trade-off between generating sufficient final mass for library construction and minimizing PCR amplification artifacts. The number of cDNA cycles should also be reduced if large numbers of cells are sampled.

(4) The product can be stored at 4°C for up to 72 hours or at −20°C for up to a week, or proceed to the next step.

**4.3 cDNA cleanup-SPRIselect timing: 50 minutes**

(1) Vortex to resuspend the SPRIselect reagent. Add 60 µL SPRIselect reagent (0.6×) to each sample and pipette set to 150 µL to mix 15 times.
(2) Incubate 5 minutes at room temperature and place on the magnet•High until the solution clears.
(3) Transfer 75 µL supernatant to a new tube strip without disturbing the pellet and maintain at room temperature.

**4.3.1 Pellet cleanup (for gene expression library) timing: 30 minute-STOP POINT**

(1) Add 200 µL 80% ethanol to the pellet, wait 30 seconds, and remove the ethanol.
(2) Repeat step 1 for a total of 2 washes, centrifuge briefly, and place on the magnet•Low.
(3) Remove any remaining ethanol and air-dry for 2 minutes.

**Caution:** Do not exceed 2 minutes, as this will decrease elution efficiency.

(4) Remove from the magnet, add 40.5 µL buffer EB, and pipette mix 15 times. Incubate 2 minutes at room temperature.
(5) Place the tube strip on the magnet•High until the solution clears, transfer 40 µL sample to a new tube strip and store at 4°C for up to 72 hours or at −20°C for up to 4 weeks, or proceed to step 4.4 followed by step 5 for gene expression library construction.

**Figure 4.**
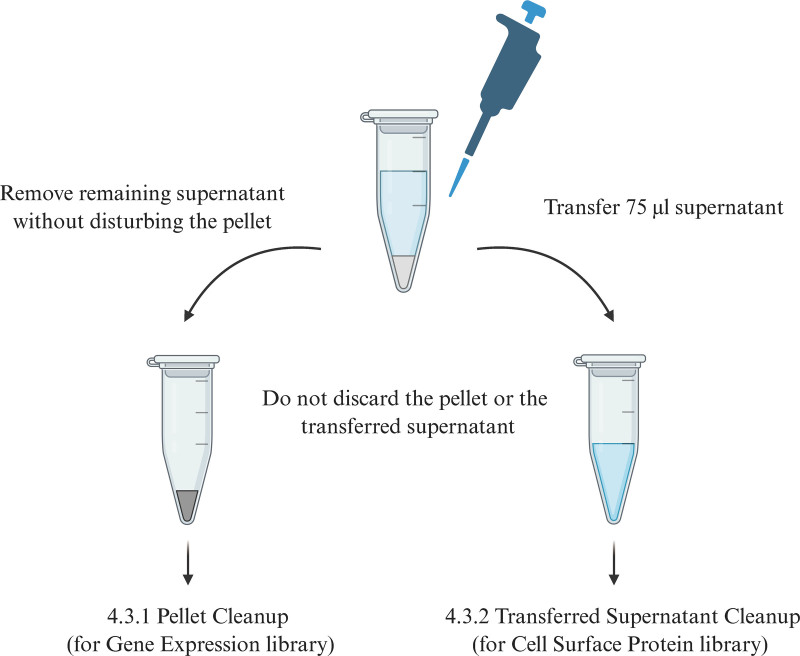
cDNA Cleanup-SPRIselect overview. cDNA = complementary deoxyribonucleic acid.

**4.3.2 Transferred supernatant cleanup (for cell surface protein library) (carried out in parallel with 4.3.1, see Fig. 4) timing: 20 minute-STOP POINT**

(6) Vortex to resuspend the SPRIselect reagent. Add 70 µL SPRIselect reagent (2.1×) to 75 µL of the transferred supernatant and pipette set to 150 µL to mix 15 times.
(7) Incubate for 5 minutes at room temperature and place on the magnet•High until the solution clears.
(8) Remove supernatant and add 200 µL 80% ethanol to the pellet. Wait 30 seconds and remove the ethanol.
(9) Repeat step 3 for a total of 2 washes, centrifuge briefly, and place on the magnet•Low.
(10) Remove any remaining ethanol and air-dry for 2 minutes.
(11) Remove from the magnet, add 40.5 µL buffer EB, and pipette mix 15 times. Incubate 2 minutes at room temperature.
(12) Place the tube strip on the magnet•High until the solution clears, transfer 40 µL sample to a new tube strip and store at 4°C for up to 72 hours or at −20°C for up to 4 weeks, or proceed to step 6 for cell surface protein library construction.

**4.4 Post-cDNA amplification QC timing: 50 minutes**

Run 1 µL of sample from Pellet Cleanup (step 4.3.1) diluted 1:10 on an Agilent Bioanalyzer High Sensitivity chip to detect cDNA concentration.

**Caution:** For input cells with low RNA content (<1 pg total RNA/cell), 1 µL undiluted product may be run.

Lower molecular weight product (35–150 bp) may be present. This is normal and does not affect sequencing or application performance.

Alternate Quantification Methods: Agilent TapeStation/LabChip.

**5. Gene expression library construction**

**5.1 Fragmentation, end repair, and A-tailing timing: 50 minutes**

(1) Prepare a thermal cycler with the following incubation protocol:

**Table:** 

Lid temperature	Reaction volume	Run time
65°C	50 µL	~35 min
Step	Temperature	Time
Pre-cool block	4°C	Hold
Fragmentation	32°C	00:05:00
End repair and A-tailing	65°C	00:30:00
	4°C	Hold

1. Vortex Fragmentation Buffer and verify there is no precipitate. Prepare fragmentation mix on ice as follows, pipette mix and centrifuge briefly:

**Table:** 

Fragmentation mix	Fragmentation buffer	Fragmentation enzyme	Total
1× (µL)	5	10	15

(3) Transfer 10 µL purified cDNA sample from Pellet Cleanup (step 4.3.1) to a tube strip, add 25 µL Buffer EB and 15 µL fragmentation mix to each sample.

**Caution:** Note that only 10 µL (25%) cDNA sample transfer is sufficient for generating gene expression library. The remaining 30 µL (75%) cDNA sample can be stored at 4°C for up to 72 hours or at −20°C for up to 4 weeks for generating additional gene expression libraries.

(4) Pipette set to 35 µL to mix 15 times on ice and centrifuge briefly. Transfer into the pre-cooled thermal cycler (4°C) and press “SKIP” to initiate the reaction.

**5.2 Post-fragmentation, end repair, and A-tailing double-sided size selection-SPRIselect timing: 30 minutes**

(1) Vortex to resuspend SPRIselect reagent, add 30 µL SPRIselect (0.6×) reagent to each sample, and pipette set to 75 µL to mix 15 times.
(2) Incubate 5 minutes at room temperature and place on the magnet•High until the solution clears.
(3) Transfer 75 µL supernatant to a new tube strip and vortex to resuspend SPRIselect reagent. Add 10 µL SPRIselect reagent (0.8×) to each transferred supernatant and pipette set to 80 µL to mix 15 times.
(4) Incubate 5 minutes at room temperature, place on the magnet•High until the solution clears and remove 80 µL supernatant.
(5) Add 125 µL 80% ethanol to the pellet and wait 30 seconds.
(6) Remove the ethanol and repeat step 5 for a total of 2 washes, centrifuge briefly, place on the magnet•Low until the solution clears and remove remaining ethanol.
(7) Remove from the magnet, add 50.5 µL Buffer EB to each sample and pipette set to 45 µL to mix 15 times.
(8) Incubate 2 minutes at room temperature, place on the magnet•High until the solution clears and transfer 50 µL sample to a new tube strip.

**5.3 Adaptor ligation timing: 25 minutes**

(1) Prepare adaptor ligation mix. Pipette mix and centrifuge briefly:

**Table:** 

Adaptor ligation mix	Ligation buffer	DNA ligase	Adaptor oligos	Total
1× (µL)	20	10	20	50

(2) Add 50 µL Adaptor Ligation Mix to 50 µL sample, pipette set to 90 µL to mix 15 times and centrifuge briefly.
(3) Incubate in a thermal cycler with the following protocol:

**Table:** 

Lid temperature	Reaction volume	Run time
30°C	100 µL	15 min
Step	Temperature	Time
1	20°C	00:15:00
2	4°C	Hold

**5.4 Post-ligation cleanup-SPRIselect timing: 30 minutes**

(1) Vortex to resuspend SPRIselect Reagent, add 80 µL SPRIselect Reagent (0.8×) to each sample and pipette set to 150 µL to mix 15 times.
(2) Incubate 5 minutes at room temperature, place on the magnet•High until the solution clears and remove the supernatant.
(3) Add 200 µL 80% ethanol to the pellet, wait 30 seconds, and remove the ethanol.
(4) Repeat step 3 for a total of 2 washes, centrifuge briefly, and place on the magnet•Low.
(5) Remove any remaining ethanol and air-dry for 2 minutes.
Remove from the magnet, add 30.5 µL Buffer EB, and pipette mix 15 times.
(6) Incubate 2 minutes at room temperature, place on the magnet•Low until the solution clears and transfer 30 µL sample to a new tube strip.

**5.5 Sample index PCR timing: 40 minute-STOP POINT**

**Caution:** Choose the appropriate sample index sets to ensure that no sample indices overlap in a multiplexed sequencing run. Record the Sample Index name used.

(7) Add 50 µL Amp Mix to 30 µL sample, add 20 µL of an individual Sample Index to each sample and record the well ID used, pipette set to 90 µL to mix 5 times, and centrifuge briefly.
(8) Incubate in a thermal cycler with the following protocol:

**Table:** 

Lid temperature	Reaction volume	Run time
105°C	100 µL	~25–40 min
Step	Temperature	Time
1	98°C	00:00:45
2	98°C	00:00:20
3	54°C	00:00:30
4	72°C	00:00:20
5	Go to step 2, check Table 5.5.1 for the number of cycles	
6	72°C	00:01:00
7	4°C	Hold

**Table 5.5.1:** Recommended cycle number optimization

cDNA input	Total cycles
0.25–25 ng	14–16
25–150 ng	12–14
150–500 ng	10–12
500–1000 ng	8–10
1000–1500 ng	6–8
>1500 ng	5

**Caution:** The total cycles should be optimized based on 25% carry-forward cDNA yield/input calculated during cDNA QC (step 4.4).

(9) The product can be stored at 4°C for up to 72 hours or proceed to the next step.

**5.6 Post-sample index PCR double-sided size selection-SPRIselect timing: 30 minute-STOP POINT**

(1) Vortex to resuspend the SPRIselect reagent, add 60 µL SPRIselect Reagent (0.6×) to each sample, and pipette set to 150 µL to mix 15 times.
(2) Incubate 5 minutes at room temperature, place the magnet•High until the solution clears and transfer 150 µL supernatant to a new tube strip.
(3) Vortex to resuspend the SPRIselect reagent, add 20 µL SPRIselect Reagent (0.8×) to each transferred supernatant and pipette set to 150 µL to mix 15 times.
(4) Incubate 5 minutes at room temperature, place the magnet•High until the solution clears and remove 165 µL supernatant.
(5) Add 200 µL 80% ethanol to the pellet, wait 30 seconds, and remove the ethanol.
(6) Repeat step 5 for a total of 2 washes, centrifuge briefly, place on the magnet•Low and remove remaining ethanol.
(7) Remove from the magnet, add 35.5 µL Buffer EB, and pipette mix 15 times.
(8) Incubate 2 minutes at room temperature, place on the magnet•Low until the solution clears and transfer 35 µL to a new tube strip.
(9) Store at 4°C for up to 72 hours or at −20°C for long-term storage.

**Figure 5.**
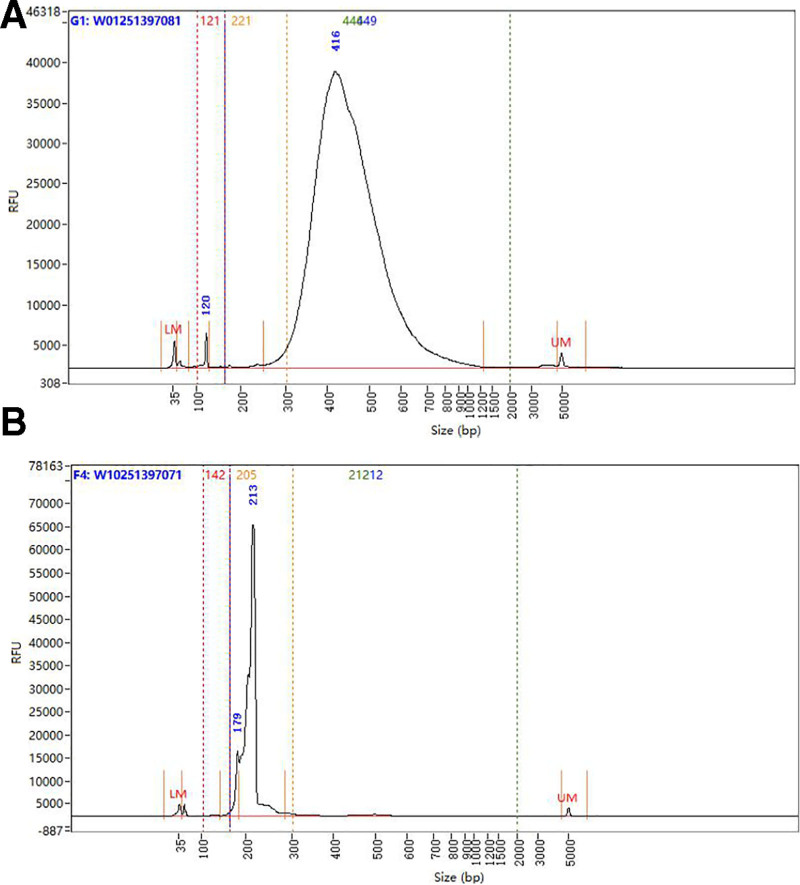
The representative traces of library construction. (A) Fragment size distribution of a high-quality gene expression library. (B) Representative fragment distribution from a standard cell surface protein library.

**5.7 Post-library construction QC timing: 50 minutes (Fig. 5**)

Run 1 µL sample at 1:10 dilution on an Agilent Bioanalyzer High Sensitivity chip, select the region between 200 and 2000 bp to determine the average fragment size from the Bioanalyzer trace. This will be used as the insert size for library quantification.

If additional peaks below 200 bp are observed, repeat step 5.6 Post sample index PCR double-sided size selection-SPRIselect. Add nuclease-free water to bring the library volume to 100 µL before performing it and note that about 40% of material may be lost.

Alternatively, libraries that will be sequenced together can first be pooled and then used as input into step 5.6.

Alternate Quantification Methods: Agilent TapeStation/LabChip.

**6. Cell surface protein library construction**

**6.1 Sample index PCR timing: 40 minutes**

**Caution:** Choose the appropriate sample index sets to ensure that no sample indices overlap in a multiplexed sequencing run. Record the Sample Index name used.

(1) Prepare sample index PCR mix as follows:

**Table:** 

Sample index PCR mix	Amp mix	Buffer EB	Total
1× (µL)	50	25	75

(2) Transfer 5 µL DNA sample from the Transferred Supernatant Cleanup (step 4.3.2) to a new tube strip.

**Caution:** Note that only 5 µL DNA sample is sufficient for generating cell surface protein library. The remaining 35 µL DNA sample can be stored at 4°C for up to 72 hours or at −20°C for up to 4 weeks for generating additional cell surface protein libraries.

(3) Add 75 µL sample index PCR mix to each sample, add 20 µL of Sample Index to each sample and record their assignment.
(4) Pipette set to 90 µL to mix 5 times and centrifuge briefly.
(5) Incubate in a thermal cycler with the following protocol:

**Table:** 

Lid temperature	Reaction volume	Run time
105°C	100 µL	~25–40 min
Step	Temperature	Time
1	98°C	00:00:45
2	98°C	00:00:20
3	54°C	00:00:30
4	72°C	00:00:20
5	Go to step 2, repeat 9 times for a total of 10 cycles	
6	72°C	00:01:00
7	4°C	Hold

**Caution:** Optimization of cycle number may be needed based on target protein expression levels and number of antibodies used for labeling.

**6.2 Post-sample index PCR size selection-SPRIselect timing: 20 minute-STOP POINT**

(1) Vortex to resuspend the SPRIselect reagent, add 120 µL SPRIselect Reagent (1.2×) to each sample and pipette set to 150 µL to mix 15 times.
(2) Incubate 5 minutes at room temperature, place on the magnet•High until the solution clears and remove the supernatant.
(3) Add 300 µL 80% ethanol to the pellet, wait 30 seconds, and remove the ethanol.
(4) Add 200 µL 80% ethanol to the pellet, wait 30 seconds, and remove the ethanol.
(5) Centrifuge briefly, place on the magnet•Low and remove remaining ethanol.
(6) Remove from the magnet, add 40.5 µL Buffer EB, and pipette mix 15 times.
(7) Incubate 2 minutes at room temperature, place on the magnet•Low until the solution clears and transfer 40 µL to a new tube strip.
(8) The product can be stored at 4°C for up to 72 hours or at −20°C for long-term storage.

**6.3 Post-library construction QC timing: 50 minutes (Fig. 5**)

Run 1 µL sample at 1:10 dilution on an Agilent Bioanalyzer High Sensitivity chip, select the region between 150 and 300 bp to determine the average fragment size from the Bioanalyzer trace, and this will be used as the insert size for library quantification.

Alternate Quantification Methods: Agilent TapeStation/LabChip.

**7. Sequencing**

A. Sequencing libraries

Single Cell Gene Expression and Cell Surface Protein Dual Index libraries comprise standard paired-end constructs which begin with P5 and end with P7. These libraries include Cell Barcodes at the start of Read 1 and Read 1N, respectively, while i7 and i5 sample index sequences are incorporated as the sample index read. Read 1 and Read 2 are standard sequencing primer sites used in paired-end sequencing of Single Cell Gene Expression libraries. Read 1N and Read 2 are used for paired-end sequencing of Single Cell Cell Surface Protein libraries.

B. Sample indices

If multiple samples are pooled in a sequencing lane, the sample index name is needed in the sample sheet used for generating FASTQs with “cellranger mkfastq.” Samples utilizing the same sample index should not be pooled together or run on the same flow cell lane, as this would not enable correct sample demultiplexing.

C. Gene expression library sequencing depth and run parameters

**Table:** 

Sequencing depth	Minimum 20,000 read pairs per cell
Sequencing type	Paired-end, dual indexing
Sequencing read	Recommended number of cycles
Read 1	28 cycles
i7 Index	10 cycles
i5 Index	10 cycles
Read 2	90 cycles

1. D. Cell surface protein library sequencing depth and run parameters

**Caution:** Pooling single-cell gene expression and cell surface protein dual index libraries is recommended for sequencing to maintain nucleotide diversity.

**Table:** 

Sequencing depth	Minimum 5000 read pairs per cell
Sequencing type	Paired-end, dual indexing
Sequencing read	Recommended number of cycles
Read 1	28 cycles
i7 Index	10 cycles
i5 Index	10 cycles
Read 2	90 cycles

E. Library pooling

The gene expression and the cell surface protein libraries may be pooled for sequencing, taking into account the differences in cell number and per-cell read depth requirements between each library. Samples utilizing the same sample index should not be pooled together or run on the same flow cell lane, as this would not enable correct sample demultiplexing.

F. Data analysis and visualization

Sequencing data may be analyzed using Cell Ranger and visualized using Loupe Browser.

Key features are listed below.

Cell Ranger^22^: Cell Ranger is a set of analysis pipelines that processes single gene expression data to align reads, generate feature barcode matrices, and perform clustering and gene expression analysis.

- Input: Base call (BCL) and FASTQ
- Output: BAM, MEX, CSV, HDF5, Web Summary, loupe
- Operating System: Linux

Loupe Browser^23^: Loupe Browser is an interactive data visualization tool that requires no prior programming knowledge.

- Input: .cloupe
- Output: Data visualization, including t-SNE and UMAP projections, custom clusters, differentially expressed genes
- Operating System: MacOS, Windows

## 4. TROUBLESHOOTING

**4.1 Sample preparation:**

Cell viability is one of the key factors influencing the quality and quantity of output. Increase BSA/FBS concentration in wash and resuspension buffers (up to 10% FBS) to maintain cell viability and minimize cell loss. If cell viability cannot be maintained in a PBS-based buffer, it is possible to wash and resuspend in common cell culture media plus BSA/FBS (1% BSA or up to 10% FBS).

**4.2 Analysis of the antibody-oligo conjugate**

**Figure:**
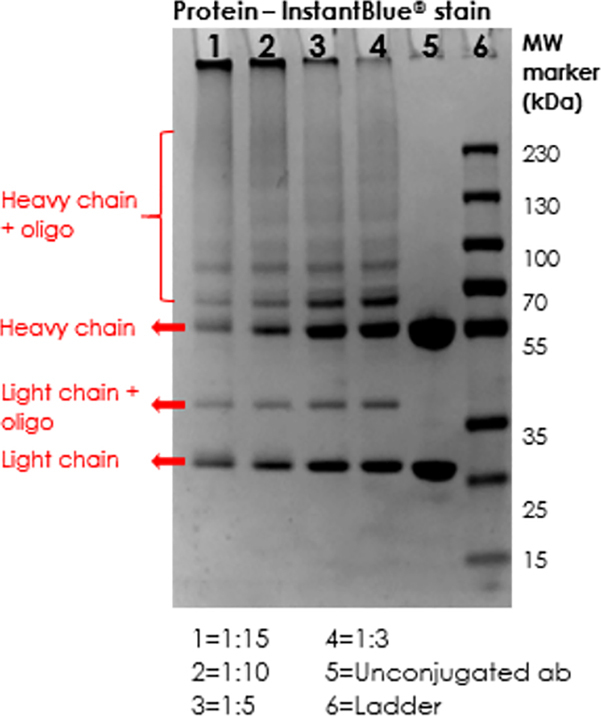


**Caution:** IgG is composed of 2 heavy chains and 2 light chains. Not all chains will be linked to an oligonucleotide. Even if the conjugation is excellent, there will still be a large number of unlabeled antibody heavy chains and light chains. Antibodies with a high conjugation ratio often undergo cross-linking, which hinders the migration of the conjugated antibodies on the gel.

The magnitude of the heavy chain displacement depends on the size of the oligonucleotide to which it is conjugated. The larger the oligonucleotide, the larger the resulting band, and vice versa.

Other antibody subtypes will generate a different banding pattern on the gel.

**4.3 Prepare antibody mix supernatant**

This protocol uses an antibody-to-oligonucleotide ratio of 1:3 for conjugation. Optimization may be required depending on the antibody used. And to assess conjugation efficiency, the BCA or Bradford protein assay kit may be employed to determine the final concentration of the conjugated antibody.

**4.4 Cell washing (for samples with <70% viable cells**)

If enrichment of certain rare populations is desired, this protocol can be further modified that include only a single washing step, though FACS must be performed at this stage for enrichment of labeled and viable cells.

**4.5 GEM-RT incubation**

After Chip is removed from the machine and the wells are exposed, consistent liquid volume and clarity across all the wells indicate successful GEM formation. If the liquid in some wells is notably clearer or has a obviously smaller volume than others, this signifies a reagent clog and wetting failure has occurred.

## ACKNOWLEDGMENTS

This work was supported by the Chinese Academy of Medical Sciences (CAMS) Innovation Funds for Medical Sciences (2024-I2M-3-001).

We want to express our gratitude for the drawing materials provided by BioRender. The antibody-oligonucleotide conjugation step follows the manufacturer’s instructions provided by Abcam and the commercial assays were produced by Anoroad.

## AUTHOR CONTRIBUTIONS

C.C. conceived the study; S.H. designed and performed experiments, analyzed and interpreted data, and wrote the manuscript; S.L. performed additional experiments; C.C. supervised the study and wrote the manuscript; all authors approved the final version of the manuscript.
